# Both rare and common genetic variants contribute to autism in the Faroe Islands

**DOI:** 10.1038/s41525-018-0075-2

**Published:** 2019-01-21

**Authors:** Claire S Leblond, Freddy Cliquet, Coralie Carton, Guillaume Huguet, Alexandre Mathieu, Thomas Kergrohen, Julien Buratti, Nathalie Lemière, Laurence Cuisset, Thierry Bienvenu, Anne Boland, Jean-François Deleuze, Tormodur Stora, Rannva Biskupstoe, Jónrit Halling, Guðrið Andorsdóttir, Eva Billstedt, Christopher Gillberg, Thomas Bourgeron

**Affiliations:** 1 Human Genetics and Cognitive Functions Unit, Institut Pasteur, Paris, France; 2 CNRS UMR 3571 Genes, Synapses and Cognition, Institut Pasteur, Paris, France; 3 University Paris Diderot, Sorbonne Paris Cité, Paris, France; 4 Centre de Bioinformatique, Biostatistique et Biologie Intégrative, Paris, France; 5 Laboratoire de Génétique et Biologie Moléculaires, Hôpital Cochin, HUPC, Paris, France; 6 INSERM U894, Institut de Psychiatrie et de Neurosciences de Paris, Paris, France; 7 Centre National de Recherche en Génomique Humaine (CNRGH), Institut de Biologie François Jacob, CEA, Université Paris-Saclay, Evry, France; 8 Department of Psychiatry, National Hospital Faroe Islands, Tórshavn, Faroe Islands; 9 Ministry of Health Genetic Biobank of the Faroes Tórshavn Faeroe Islands, Tórshavn, Faroe Islands; 10 Faculty of Science and Technology, The University of the Faroe Islands, Tórshavn, Faroe Islands; 11 Gillberg Neuropsychiatry Centre, Institute of Neuroscience and Physiology, Gothenburg University, Gothenburg, Sweden; 12 University of Glasgow, Glasgow, Scotland UK

## Abstract

The number of genes associated with autism is increasing, but few studies have been performed on epidemiological cohorts and in isolated populations. Here, we investigated 357 individuals from the Faroe Islands including 36 individuals with autism, 136 of their relatives and 185 non-autism controls. Data from SNP array and whole exome sequencing revealed that individuals with autism had a higher burden of rare exonic copy-number variants altering autism associated genes (deletions (*p* *=* 0.0352) or duplications (*p* *=* 0.0352)), higher inbreeding status (*p* *=* 0.023) and a higher load of rare homozygous deleterious variants (*p* *=* 0.011) compared to controls. Our analysis supports the role of several genes/loci associated with autism (e.g., *NRXN1*, *ADNP*, 22q11 deletion) and identified new truncating (e.g.*, GRIK2*, *ROBO1, NINL*, and *IMMP2L*) or recessive deleterious variants (e.g.*, KIRREL3* and *CNTNAP2*) affecting autism-associated genes. It also revealed three genes involved in synaptic plasticity, *RIMS4*, *KALRN*, and *PLA2G4A*, carrying de novo deleterious variants in individuals with autism without intellectual disability. In summary, our analysis provides a better understanding of the genetic architecture of autism in isolated populations by highlighting the role of both common and rare gene variants and pointing at new autism-risk genes. It also indicates that more knowledge about how multiple genetic hits affect neuronal function will be necessary to fully understand the genetic architecture of autism.

## Genetic correlates of autism in natives of remote islands

A study of the genetic architecture of autism among people living on the remote Faroe Islands highlights the role of both common and rare gene variants to autism. Claire Leblond from the Institut Pasteur in Paris, France, and colleagues profiled the genetics of 357 Faroese individuals, including 36 with autism, 136 of their relatives and 185 non-autistic controls. Similar to the findings of genetic studies of autism from elsewhere, the researchers discovered rare structural variants in known autism-associated genes and a few new candidate genes linked to brain function. However, unlike studies from larger mainland populations, they also showed that inbreeding on these remote islands increased the likelihood of carrying two copies of the point mutations that contribute to autism.

## Introduction

Autism spectrum conditions (ASCs; henceforth ‘autism’) are diagnosed in 1–2% of the population worldwide and are characterized by atypical social communication and the presence of restricted interests, as well as stereotyped and repetitive behaviors. Individuals with autism can also suffer from other conditions including intellectual disability (ID), attention-deficit hyperactivity disorder (ADHD), anxiety, depression, epilepsy, motor control difficulties, tics, sleep disorders, or gastrointestinal problems.^1^ The heritability of autism is very high^2^ and molecular genetic studies revealed that the genetic risk for autism is shaped by a combination of rare and common variants.^3–5^ Thus, the genetic susceptibility to autism can vary from one individual to another. In some cases, a single de novo causative variant can be detected. On the contrary, in some cases, the genetic architecture is more complex and involves thousands of common genetic variants, each one with low impact but collectively increasing the susceptibility to autism. Most of our knowledge on the genetics of autism comes from studies on unrelated individuals with autism who do not share a recent common ancestor. Several studies investigated families with autism from countries where consanguinity is high,^6^ but the genetic architecture of autism in isolated populations remains largely unknown.

**Fig. 1:**
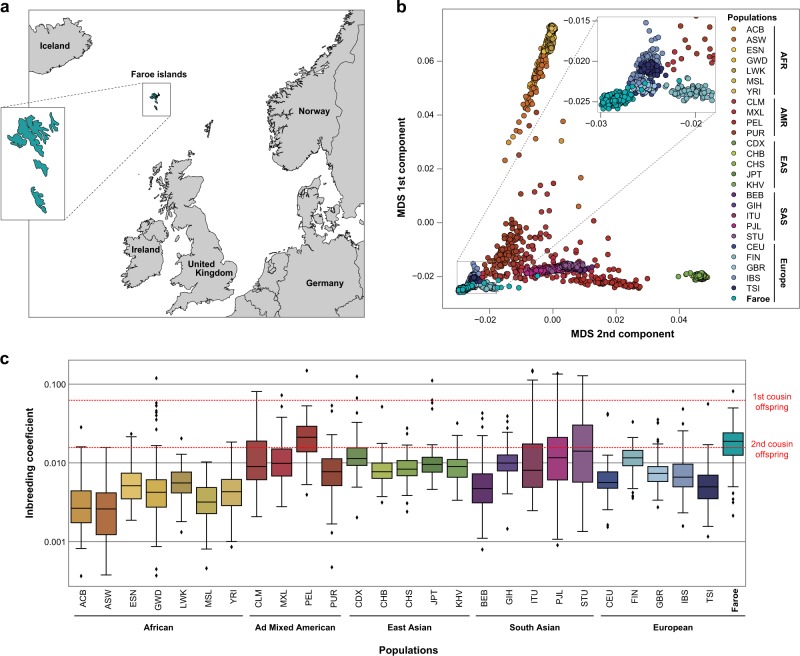
Genetic background of the Faroese population. **a** Geographic localization of the Faroe Islands. **b** Multidimensional scaling plots (MDS) of genome-wide identity by state (IBS) pairwise distances between 1000 Genomes and Faroese populations. Each dot represents an individual and the distance between two dots corresponds to genetic distance based on genome-wide pairwise IBS calculations. **c** Degree of inbreeding across 1000 Genomes and Faroese populations. The inbreeding coefficients of the Faroe non-autism control individuals (*n* = 176) were compared to the 1000 Genomes populations. The 26 populations from 1000 Genomes project are described in S1 Appendix. Logarithmic scale was used for *y*-axis

The Faroe Islands is an archipelago located in the North Atlantic Ocean, halfway between Norway, Iceland, and Scotland (Fig. 1a). The population (approximately 49,000 inhabitants) was founded in the 9th century by a small number of emigrants from Norway. The population remained at a small size for centuries until it experienced a rapid expansion in the 1800s. Previous genetic studies indicated that individuals from Scotland, Norway, Sweden, Ireland, Iceland, and British Isles have significantly contributed to the current gene pool of the Faroese population.^7,8^

We previously showed that the prevalence of autism in the Faroe Islands (0.94% of the population^9–11^) was similar to many other western countries. In this study, we ascertained the genetic profile of 357 individuals including an epidemiological cohort of 36 individuals with autism born between 1985 and 1994 (Fig. 1 and S1 Fig), their relatives (*n* = 136) and a group of 185 controls. We first investigated the known causes of autism and then identified new candidate genes. We also estimated the impact of inbreeding and the load of deleterious homozygous variants on the risk of autism. Finally, both rare and common genetic variants were used to stratify individuals with autism and to compare their genetic and clinical profiles.

## Results

### The genetic diversity in the Faroe Islands

A total of 67 children and adolescents with autism were detected in a total population study of individuals aged 8–17 years living in the Faroe Islands and born between 1985 and 1994.^9,10^ Thirty-six of the 67 individuals (54% of the total group) signed (or had parents who signed) informed consent forms and were included in the genetic study. Participants and non-participants in the genetic study were similar in terms of gender and cognitive abilities (S1 Fig). In addition, we collected DNA from 136 of their relatives and from 185 “non-autism” controls. The genetic profile included a high-density Illumina SNP array interrogating >4.3 millions of single nucleotide polymorphisms (SNPs) and a whole exome sequencing (WES) to discover new variants. Using identical-by-state (IBS) genomic distance (see Methods), we first compared the population structure of individuals from the Faroe Islands with worldwide populations (Fig. 1b). All individuals were clustered in the Faroese population with the exception of seven controls who were removed from the analyses. Using admixture, we showed that individuals from the Faroe Islands have their genome in majority constituted from “European component” (S2 Fig). As expected from the demographic history, individuals from the Faroese population displayed a higher degree of inbreeding compared with other world populations (Fig. 1c and S3 Fig). The only population from the 1000 genomes project that was more inbreed than the Faroese population was the Peruvian from Lima.

### Contribution of de novo variants

We ascertained the burden of de novo variants since they are key players in the genetics of autism.^12^ The de novo variants were identified for 31 independent families including 28 individuals with autism and 45 siblings for whom DNA of both parents was available (see Clinical notes in S1 Appendix for the pedigrees). The combined analysis of genotyping and WES data revealed the presence of de novo chromosomal abnormalities and exonic copy-number variants (CNVs) in 3 out of 26 individuals with autism (11.5%) and 1 out of 43 siblings (2.3%). One female PN400129 had a trisomy of chromosome 21 and was diagnosed with autism, ID and Down syndrome (S4 Fig and S1 Table). One female PN400533 with atypical autism without ID carried a de novo 2.9 Mb deletion on chromosome 22 causing 22q11.2 deletion syndrome (also known as DiGeorge/VeloCardioFacial syndrome). A male PN400115 with atypical autism without ID carried a de novo 425.5 kb deletion removing the first six exons of the *NRXN1α*. We also found a 91.4 kb deletion removing all exons of *ADNP* in a male with autism and ID (PN400125). The deletion was not found in the mother and was most likely de novo, but father’s DNA was not available and none of the SNPs within the deletion were informative to confirm the de novo status of the deletion. The de novo CNV observed in a sibling (PN400170) was a duplication of 782 kb affecting 5 genes (*CNPY1*, *DPP6*, *EN2*, *HTR5A*, *INSIG1* and *PAXIP1*).

Using the WES data, we detected the presence of de novo single nucleotide variants (SNVs) and small insertions/deletions (indels)(S2 Table). Overall, the rate of de novo exonic SNV/indels was similar to other studies^13^ and was not different in individuals with autism (0.93) and their siblings (0.96). The variants were considered as probably deleterious when they were likely gene disruptive (LGD, for example stop gain or frame shift variant) or missense events with a combined annotation dependent depletion (CADD) score > 30 (MIS30).^14^ There was also no significant increase in the rate of de novo deleterious variants in individuals with autism compared to their siblings (two-sided Fisher’s exact test: 9/31 (29%) MIS30 + LGD SNV in autism, 6/49 (12%) MIS30 + LGD SNV in sibling; *p* = 0.08, OR = 2.89, CI 95% [0.8–11.25]) and no significant enrichment in genes associated with autism (SFARI genes^15^) or expressed in the brain (Brain genes, see methods for gene selection). Nevertheless, several deleterious variants were identified in known genes for autism (*MECP2*) or compelling candidate genes (*RIMS4, KALRN, PLA2G4A)* (S5 Fig). Clinical details on the individuals with autism carrying those variants are available in the S1 Appendix.

### Contribution of rare CNVs and SNVs/indels variants

**Fig. 2:**
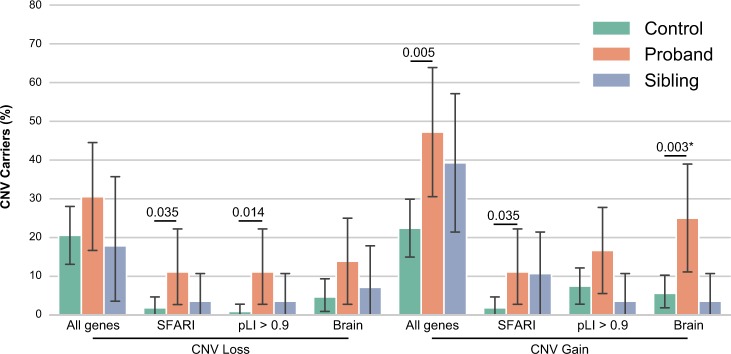
Rare CNVs in Faroese individuals. Rare copy-number variant (CNV) analysis among gene-set lists within Faroe individuals (XHMM CNV calling from WES and CNV frequency < 0.01 in controls). The number of exonic CNV carriers altering any gene or gene-set lists (SFARI genes, pLI > 0.9 genes and Brain genes, see Materials and Methods section) were compared between individuals with autism, siblings and controls (one-sided Fisher’s exact test: *n*_autism _= 36, *n*_sib_ = 28, *n*_controls _= 107, *p*_CNV_loss_SFARI _= 0.035, OR_CNV_loss_All_SFARI _= 6.56; *p*_CNV_loss_pLI>0.9 _= 0.014, OR_CNV_loss_pLI>0.9 _= 13.25; *p*_CNV_gain_All_genes _= 0.005, OR_CNV_gain_All_genes _= 3.09; *p*_CNV_gain_SFARI _= 0.035, OR_CNV_gain_All_SFARI _= 6.56; *p*_CNV_gain_Brain _= 0.003*, OR_CNV_gain_All_Brain _= 5.61; *indicates the one withstanding Bonferroni correction for 12 tests; for families with multiple siblings, only one sibling was kept (closest on age)). Error bars represent confidence interval

Global rare CNV analysis was performed using the XHMM CNV calling from WES data and the overall burden of rare exonic CNVs (frequency < 0.01) was higher in individuals with autism compared to controls for duplications (*p*_*nominal*_ *=* 0.005) (Fig. 2 and S3 Table). The burden of deletions was higher for autism-associated genes listed in the SFARI database (*p*_*nominal*_ *=* 0.035) and for genes intolerant to loss-of-function variant (pLI > 0.9)^16^ (*p*_*nominal*_ *=* 0.014). For duplications, SFARI genes or genes expressed in the brain were more frequently duplicated in individuals with autism compared to controls (“SFARI” *p*_*nominal*_ *=* 0.035 and “Brain” *p*_*nominal*_ *=* 0.003, *p*_*corrected* _*=* 0.036, 12 tests). Most of these differences however do not survive corrections for multiple tests and we had no significant difference between individuals with autism and their siblings. Among the SFARI genes affected by the CNVs, we identified a 58 kb maternal inherited deletion including the *IMMP2L*, a 2 Mb paternal inherited duplication on the pseudo-autosomal region 1 including *SHOX* and *ASMT*, and a 39 kb maternal inherited duplication of *TBL1XR1* (S3 Table).

Our global analysis was restricted to CNVs affecting exons, but using CNV called from SNP dataset we identified an additional large 357 kb duplication within intron 5 of the *NLGN1* gene and covering a long *NLGN1* antisense noncoding RNA that was paternally inherited in a female (PN400102) with autism and no ID. There was no such rare intronic CNVs in the SFARI genes in siblings and controls.

We then run single variant and gene-wise association tests from rare SNV/indels (MAF < 5%) and none of associations were genome-wide or gene-wide significant (S6-7 Fig and S4-S7 Tables). We found truncating variants affecting several SFARI genes (e.g., *PRODH*, *ERBB4*, *GRIK2*, *ROBO1*, *RBMS3*, and *IMMP2L*; S6 Fig. and S7 Table), but the number of patients carrying these variants was too low to detect a significant association.

### Founder effect

As the Faroe population originates from a small number of founders, specific changes in allele frequencies through genetic drift could contribute to the risk of autism. In order to detect a founder effect, we investigated the contribution of the common variants (MAF > 5%) using genome wide association studies (GWAS) in cases and controls using three models (allelic, recessive and dominant) and a burden/collapsing test that aggregates the variants located in a gene. Neither GWAS nor gene-based analysis revealed a specific variant that pass genome wide significance. The absence of a statistically significant variant shared by the patients indicates that, within this sample, we could not detect a founder effect for the genetic risk of autism in the Faroe Island. The results of the GWAS and gene-based analysis are presented in S6 and S8-9 Figs and S9-S11 Tables.

### Contribution of recessive variants

**Fig. 3:**
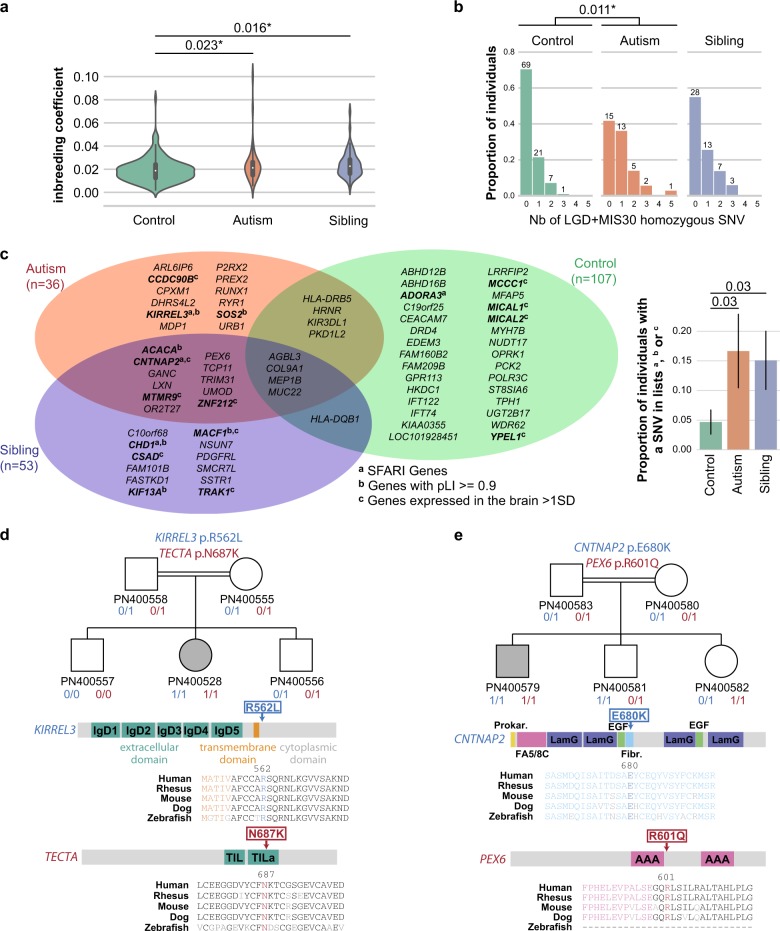
Genetic recessive mutations in Faroese individuals with autism. **a** Distribution of the inbreeding coefficient in Faroese individuals (one-sided Mann Whitney *U*-test: *n*_autism _= 36, *n*_control _= 176, *n*_sibling _= 30; *U*_control.vs.autism _= 2500, *p*_control.vs.autism _= 0.023; *U*_control.vs.sibling _= 1995, *p*_control.vs. sibling _= 0.016; sibling inbreeding coefficients were averaged out by family; *P*-values were adjusted for principal component ancestry (3 principal components); *indicates the one withstanding Bonferroni correction for two paired comparisons). **b**. Number of rare LGD + MIS30 homozygous mutations carried per individual (one-sided Mann Whitney U-test: n_autism _= 36, *n*_control _= 107, *n*_sibling _= 28; *U*_control.vs.autism _= 1305, *p*_controls.vs.autisms _= 0.011; sibling number of rare SNV were averaged out by family; P-values were adjusted for inbreeding; * indicates the one withstanding Bonferroni correction for two paired comparisons). **c** Venn diagram of the genes carrying the variants from **b**. Genes names are in bold and annotated when they are part of our gene-set lists (SFARI genes, pLI > 0.9 genes and Brain genes, see methods section). The plot on the right shows the proportion of individuals in each category carrying at least one mutated gene in our gene-sets lists (Fisher’s exact test: *p*_controls.vs.autisms _= 0.03; *p*_controls.vs. siblings _= 0.03). Error bars represent standard error. **d** and **e** are describing two specific families carrying multiple variants. “0” and “1” refer to wildtype or mutated allele, respectively. The localizations of the variants are indicated along the proteins and alignments throughout species showed the strong conservation of the altered amino acids. Nb Number, SNV Single Nucleotide Variant, LGD likely gene disruptive, MIS30 missense variants with CADD score ≥30, IgD immunoglobulin domain, TIL Trypsin Inhibitor-like, FA5/8 C Coagulation factor 5/8 type C domain, LamG Laminin G domain, EGF epidermal growth factor like domain, Fibr. Fibrinogen, alpha/beta/gamma chain, C-terminal globular domain, AAA ATPases associated domains

Since inbreeding increases the risk for individuals to carry homozygous deleterious variants, we first compared the inbreeding coefficient of the individuals with autism, their relatives and controls. Patients (*p* *=* 0.023), as well as their siblings (*p* *=* 0.016) had a higher inbreeding coefficient compared with controls (Fig. 3a). Remarkably, even after correction for inbreeding, we found that individuals with autism were carrying more deleterious homozygous variants (LGD, MIS30, gnomAD MAF < 1%) than controls (*p* *=* 0.011; Fig. 3b and S8 Table). Genes carrying deleterious homozygous variants in affected individuals were significantly enriched in the combined gene-set lists (SFARI + pLI > 0.9 + Brain genes) compared to controls (*p* *=* 0.03; Fig. 3c).

In one female individual PN400528 (inbreeding coefficient *F* = 0.04), we found a *KIRREL3* homozygous damaging missense variant (p.R562L, absent in gnomAD) affecting a conserved residue in the cytoplasmic domain of this synaptic adhesion molecule^17^ listed in SFARI and associated with neurodevelopmental disorders^18^ (Fig. 3d). Interestingly, this female with autism and a normal IQ was also homozygous for another deleterious variant (p.N687K, rs139165033, gnomAD European allele frequency = 0.003165) affecting *TECTA*, a SFARI gene associated with autism and deafness.^19^

In another family, the male PN400579 (inbreeding coefficient *F* = 0.1) was homozygous for two variants affecting *CNTNAP2* and *PEX6* (Fig. 3e). Recessive *CNTNAP2* variants are associated with Pitt-Hopkins like syndrome 1 and cortical dysplasia-focal epilepsy syndrome (MIM #610042). The *CNTNAP2* p.E680K variant (rs368905425, gnomAD European allele frequency = 0.0001979) affects a highly conserved amino acid within the fibrinogen domain of the protein. Recessive *PEX6* variants are associated with Heimler syndrome 2, a recessive peroxisome disorder characterized by sensorineural hearing loss, amelogenesis imperfecta and nail abnormalities, with or without visual defects (MIM #616617). The homozygous variant p.R601Q (rs34324426, gnomAD European allele frequency = 0.004849) carried by the male with autism can be considered pathogenic since it was previously detected in several independent patients diagnosed with Heimler syndrome 2.^20^ Details on the clinical profiles of the families are available in S1 Appendix.

### Estimation of the genome-wide polygenic score

**Fig. 4:**
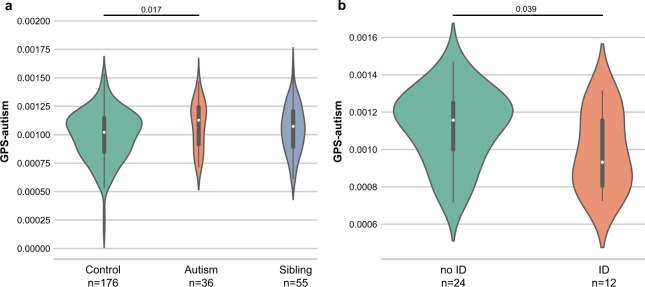
Distribution of the genome-wide polygenic score for autism in Faroese individuals. **a** Distribution of the genome-wide polygenic score for autism (GPS-autism) of controls, autisms and siblings (one-sided Mann Whitney *U*-test: *n*_autism _= 36, *n*_control _= 176, *n*_sibling _= 55; *U*_control.vs.autism _= 2460, *p*_control.vs.autism _= 0.017; Q1_autism_ = 0.0009, Q2_autism_ = 0.0011; Q3_autism_ = 0.0012, Q1_control_ = 0.0009, Q2_control_ = 0.0010, Q3_control_ = 0.0011, Q1_sibling_ = 0.0009, Q2_sibling_ = 0.0011 and Q3_sibling_ = 0.0012). **b** Distribution of the GPS-autism for the cases without intellectual disability (ID) and the cases with ID (one-sided Mann Whitney *U*-test: *n*_autism-with-ID _= 12, n_autism-without-ID _= 24; *U*_ID.vs.no-ID _= 91, *p*_ID.vs.no-ID _= 0.039; Q1_no-ID_ = 0.0010, Q2_no-ID_ = 0.0012; Q3_no-ID_ = 0.0013, Q1_ID_ = 0.0008, Q2_ID_ = 0.0009, Q3_ID_ = 0.0012). The GPS was calculated using PRSice-2 (see methods section, *P*-value threshold of 0.2 and R2 of 0.036). *P*-values were computed on data adjusted for principal component ancestry and for inbreeding

We ascertained the autism genome-wide polygenic score (GPS-autism) for each individual. The GPS-autism was calculated using PRSice-2 from a previous GWAS using over 16,000 individuals with autism^4^ who do not overlap with this sample. GPS analysis requires the estimation of a *P*-value significance threshold in order to include only variants exceeding this *P*-value threshold in the GPS calculation. Here, the best fit *P*-value threshold was 0.2 corresponding to a squared correlation coefficient (R2) of 0.036 (S10 Fig). After correction for ancestry and inbreeding, we found a significantly higher GPS-autism in individuals with autism compared to controls (*p* = 0.017; Fig. 4a). Remarkably, in the autism group, the GPS-autism was significantly higher in individuals without ID compared to those with ID (*p* = 0.039, Fig. 4b). These results should be however taken with care since our number of cases is small and we could not detect significant differences between cases and controls when *P*-value thresholds of 0.05 or 0.1 were chosen to compute the GPS.

### Genetic stratification of autism in the Faroe Islands

**Fig. 5:**
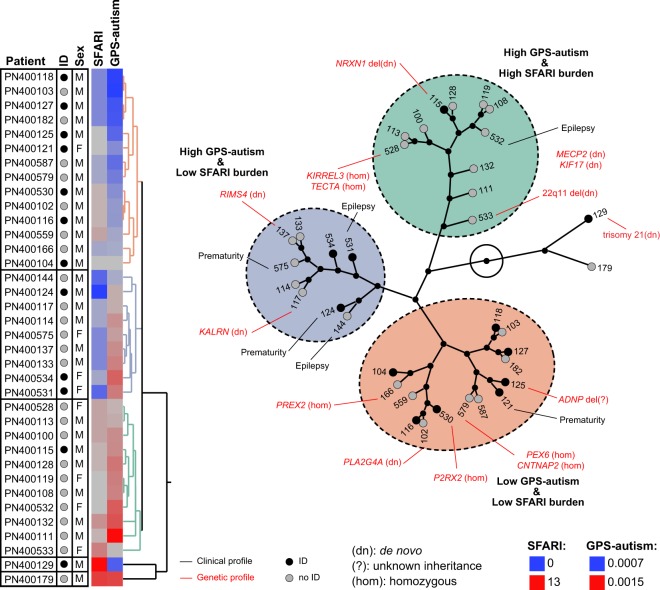
Stratification of autism in Faroese individuals. On the left, the stratification was built using hierarchical clustering on the number of genes carrying rare deleterious variants altering SFARI genes (MIS30, LGD, or CNV) and on the genome-wide polygenic score for autism (GPS-autism). The other columns were not used for the clustering. The genetic profile contains variants with a predicted impact on the condition of the individual with autism. The clinical profile gives a subset of relevant information for each individual with autism. ID intellectual disability, M male, F female, del deletion, dup duplication

In order to stratify individuals with autism, we used the number of rare deleterious variants in SFARI genes (including CNVs) and the GPS-autism estimated from the common variants (Fig. 5). This hierarchical clustering is an illustration on how similar are some patients regarding their genetic profile. We found three main clusters. The first one comprised eleven individuals with high GPS-autism and high burden of deleterious variants in SFARI genes. In this cluster, only one individual had ID (PN400115 carrying the de novo *NRXN1* deletion). In the second cluster, fourteen individuals had low GPS-autism and low burden of SFARI genes deleterious variants. In this cluster 50% of the individuals had ID. In the third cluster, nine individuals had high GPS-autism, but low burden of SFARI deleterious variants. In this cluster, 33% of the individuals had ID and it includes two individuals with autism with epilepsy and two individuals who were preterm babies.

## Discussion

In this study, we investigated a group of individuals with autism that has two distinctive features. First, the group is representative of a general population cohort of all young people living in the Faroe Islands at one point in time. Secondly, the Faroese population has a more homogeneous genetic background compared to most other populations.

We found a subset of individuals carrying strongly deleterious variants (some of which appeared de novo) affecting single genes or chromosomal regions. The chromosomal abnormalities included one trisomy 21 and one 22q11 deletion (causing Down and 22q11.2 deletion syndromes, respectively). This was not surprising to find such known genetic disorders in an epidemiologic cohort since the prevalence of autism in individuals diagnosed with these syndromes is higher than in the general population (16–37% for Down syndrome,^21^ and 23–50% for DiGeorge/VeloCardioFacial syndrome deletion^22^). We also revealed new variants in known autism-risk genes (*ADPNP*, *NRXN1*, *NINL*, *MECP2*) and identified new compelling candidate genes such as *KALRN, PLA2G4A*, and *RIMS4*.

*KALRN* codes for a guanine nucleotide exchange factor (GEF) expressed in neuronal tissue during embryonic development^23^ and has been associated with schizophrenia risk through association analysis, and, postmortem analyses of cortical *KALRN* mRNA and protein levels within individuals with autism.^24^ *KALRN* is strongly homologous to *TRIO*, a gene previously associated with autism.^25^ It is also a binding partner of the Huntingtin and a regulator of structural and functional plasticity at dendritic spines. The de novo variant (p.N2024D; CADD = 26.7) was never observed in the general population and affects a key amino acid of the GEF domain conserved through evolution. The male individual (PN400117) carrying this de novo *KALRN* variant has no ID.

*PLA2G4A* codes for the cytosolic phospholipase A2α that catalyzes the hydrolysis of membrane phospholipids to produce arachidonic acid. Mice lacking *Pla2g4a* display abnormalities in neuronal maturation (narrow synaptic cleft)^26^ and long-term potentiation. The de novo variant (p.R485C; CADD = 35) has never been observed in the general population and is predicted as a deleterious variant falling in the catalytic domain of the protein. The female PN400102 carrying this de novo variant has no ID.

*RIMS4* codes for a presynaptic protein that plays a key role in dendritic and axonal morphogenesis.^27^ *RIMS1* and *RIMS3* have already been associated with autism.^28^ The individual (PN400137) carrying the de novo *RIMS4* stop variant (p.Y204*; CADD = 38) has a normal IQ (Performance IQ = 108, Verbal IQ = 116). The variant is predicted to truncate the last quarter of the protein and was never observed in the general population. Interestingly, RIM proteins interact with voltage-dependent Ca(2+) channels (VDCCs) and suppress their activity at the presynaptic active zone to regulate neurotransmitter release. Knockdown of gamma-RIMs (*RIMS3* and *RIMS4*) attenuated glutamate release to a lesser extent than that of alpha-RIMs (*RIMS1* and *RIMS2*). As a consequence, competition between alpha- and gamma-RIMs seems to be essential for modulating the release of glutamate at the synapse. We can therefore hypothesize that the de novo *RIMS4* truncating stop variant perturbs the fine-tuning of glutamatergic release at the synapse and contributes to autism.

In genetic isolates, it is frequent to observe an increased frequency of diseases due to the presence of deleterious variants that were present in the genomes of the small group of migrants who settled the population. In the Faroe Islands, this “founder effect” was documented for several genetic diseases such as Bardet-Biedl syndrome,^29^ cystic fibrosis,^30^ 3-Methylcrotonyl-CoA carboxylase deficiency, glycogen storage disease type IIIA^31^ and retinitis pigmentosa.^32^ We confirmed that inbreeding in the Faroe Island is higher than expected compared with other populations. The median inbreeding coefficient is *F* = 0.015 ± 0.001 in the control sample and is similar to the one reported by Binzer and colleagues (2014) in their study on multiple sclerosis in the Faroe Islands (*F* = 0.018).^33^ This level of inbreeding corresponds approximately to children from parents with a second-cousin relationship (*F* = 0.016). We also observed that individuals with autism from the Faroe Islands have a significantly higher level of inbreeding and burden of recessive deleterious variants compared to their geographically matched controls. The homozygous deleterious variants carried by individuals with autism were enriched in genes included in our list of genes of interest (e.g., high intolerance for loss of function variants, expressed in the brain and previously associated with autism). However, one should note that the increased probability for having autism due to inbreeding in the Faroe Islands is relatively small (*F*_autism_ = 0.0212; *F*_controls_ = 0.0187; Cohen’s *d* = 0.43; *p* = 0.023).

In contrast to other genetic conditions, we could not detect a founder effect for autism in the Faroe Islands. Moreover, the loci identified in our study do not overlap with those detected in a previous genetic microsatellite association study in the Faroese population pointing at regions on 2q, 3p, 6q, 15q, 16p, and 18q.^34^ We also found no overlap between the variants identified in our study and those found in Faroese individuals with autism diagnosed with panic^35^ or bipolar disorders. This absence of a founder effect is also in agreement with the epidemiological observation that the prevalence of autism in the Faroese population is not higher compared to more outbred populations.

Overall, our study confirms that both rare and common genetic variants contribute to the susceptibility to autism. Although, we identified previously known genetic causes for autism and pointed at new compelling candidate genes, we also showed a contribution of the common variants illustrated by the higher GPS-autism in individuals with autism (especially those with no ID) compared to controls. To date, in the literature, very few genes are identified in individuals diagnosed with autism and intact general intelligence. Based on the genes previously reported (*NLGN3*, *NLGN4X*, duplication of *SHANK3*) and the genes found in this study (*RIMS4*, *KALRN, PLA2G4A*), it seems that the proteins involved in autism without ID converge to different parts of the post-synapse and pre-synapse rather than to pathways such as gene regulation and chromatin remodeling, but this has to be confirmed on larger cohorts. Indeed, the main limitation of our study is the small number of individuals with autism. Several LGD variants affecting autism-risk genes such as *GRIK2*^36^ or *ASMT*^37^ were found exclusively or more frequently in individual with autism compared to controls, but a replication cohort is necessary to confirm the contribution of these variants in the susceptibility to autism in the Faroe Islands.

In summary, this study improves our knowledge on the genetic architecture of autism in epidemiological cohorts and in genetic isolates by showing that the contribution of both rare and common gene variants to autism can be detected in small, but genetically homogeneous populations. It also provides new compelling candidate genes and reveals that high inbreeding and high load of homozygous deleterious variants can be a risk factor for autism. Such combined analysis investigating both rare and common gene variants might represent a useful framework to investigate, from groups to individuals, the complex genetic architecture of autism.

## Methods

### Preprint publication

The article was previously published as a preprint in bioRxiv (BIORXIV/2018/363853; 10.1101/363853).

### Ethics statement

This study was approved by the IRB of the “Institut Pasteur” of Paris (IRB00006966 Institut Pasteur, approval 2010-003). All participants over 18 and parents of the participants under 18 provided written informed consent to take part in the study (for more details see S1 Appendix).

### Genotyping

The cohort available for the genotyping is shown in Fig. 1 and S1 Fig. It includes 36 individuals with autism, 208 controls, 132 close relatives of the individuals with autism (61 siblings, 3 half-siblings and 68 parents) and 9 close relatives from the controls. DNA was extracted from blood leukocytes. The genotyping was performed at the “Centre National de Recherche en Génomique Humaine (CNRGH)” using the Infinium IlluminaOmni5-4 BeadChip (>4.3 millions of markers) from Illumina. Sample quality controls such as Sex check (based on the X chromosome homozygosity rate), Mendel errors (transmission errors within full trios) and Identity By State (IBS, see section below) were performed using PLINK 1.90.^38^

### Population genetic structure

Prior to population genetic structure analysis, the genotyping dataset of the Faroese cohort was merged to the 1000 Genomes project dataset (ftp://ftp.1000genomes.ebi.ac.uk/vol1/ftp/release/20130502/). Genotyping rate and the total number of markers after the merge was ∼ 0.93% and ∼ 4.27 millions, respectively. For the estimation of the ancestry, SNPs with genotyping call rate < 100%, failing Hardy Weinberg equilibrium test (*p* < 10^−3^) or on sex chromosomes were filtered out of the merged genotyping dataset (∼ 2.60 million markers passed filters). Genome-wide pairwise IBS calculations and Multidimensional scaling (mds) analysis on genome-wide IBS pairwise distance matrix was calculated using PLINK 1.90. IBS values have been calculated for 376 individuals from Faroe Islands and 2504 individuals from 1000 Genomes project (26 populations; see S1 Appendix) with the following calculation: 1−(0.5 × IBS1 + IBS2)/N; N is the number of tested markers; IBS1 and IBS2 are the number of markers for which one pair of individuals share either 1 or 2 identical allele(s), respectively. Out of the 376 individuals, 32 individuals were removed from further analyses, including 7 ancestry outliers in controls, 9 siblings of controls, one swap and 15 control individuals involved in pairs with IBS score higher than 0.9. For all studies, we used the first three principal components to adjust for population stratification.

For the estimation of the inbreeding coefficient, SNPs with genotyping call rate < 95%, minor allele frequency < 0.05, strong linkage disequilibrium *r* > 0.5 or failing Hardy Weinberg equilibrium test (*p* < 10^−6^) were filtered out of the Faroe SNP genotyping dataset. All homozygosity analyses were performed with Plink 1.09 on autosomes including identification of Runs Of Homozygosity (ROH) and Inbreeding coefficients calculation. For ROH detection, a threshold of 50 consecutive homozygous SNPs with a minimum density of 1 SNP/5000 kb and no minimum length was used following Gazal et al.’s guidelines.^39^ We allowed no heterozygous markers in the 50 SNPs-window. Inbreeding coefficients were calculated by estimating the proportion of the autosomal genome that is in ROH. This method was proposed by McQuillan and al (2008) and has been shown to be the most reliable, especially with small sample size.^40^ Faroe inbreeding coefficients were compared to inbreeding coefficient of the 1000 genomes project populations.

### Genome-wide association study (GWAS)

Prior to association analyses, SNPs with genotyping call rate < 90%, minor allele frequency < 0.05 or failing Hardy Weinberg equilibrium test (*p* < 10^−6^) were filtered out of the Faroe SNP genotyping dataset. The global genome wide genotyping call rate of all the individuals was superior to 90%. A total of 1,690,491 variants and 212 independent individuals (including 36 cases and 176 controls) passed these filters. Allelic, recessive and dominant GWAS were performed with Plink 1.09 using Chi-squared statistics. Manhattan and Quantile-Quantile (Q-Q) plots were generated using R. Gene and gene-set (including SFARI, pLI > 0.9 and Brain gene lists) analyses were performed with MAGMA v1.06 using principal components regression and linear regression models, respectively.

### Genome-wide polygenic score (GPS) for autism

The computation of the GPS (also named PRS for polygenic risk score) was performed with the tool PRSice2^41^ on the SNP array data using as a reference the PGC GWAS summary statistics.^4^ SNPs were not imputed since we used high density arrays (over 4 million SNPs). Briefly, GPS is calculated as a weighted sum of the number of risk alleles carried by an individual, where the risk alleles and their weights are defined by the loci and their measured effects as detected by a previous GWAS. GPS analysis requires the estimation of a *P*-value significance threshold in order to include in the GPS calculation only variants below this *P*-value threshold. For our dataset, PRSice2 with default parameters was used (except for the *P*-value threshold for which a step of 0.01 was used) and defined a *P*-value threshold of 0.2 which gives us a R^2^ (squared correlation coefficient) of 0.036 (S10 Fig).

### Whole-Exome Sequencing (WES)

Blood leukocytes DNA from 286 individuals was enriched for exonic sequences through hybridization SureSelect Human All Exon V5 (Agilent) by the CNRGH. For 67 individuals for whom the available quantity of DNA was low, we used a low-input protocol using only 200 ng of DNA compared to 3 µg for the normal protocol. The captured DNA was sequenced using a HiSeq 2000 instrument (Illumina). Coverage/depth statistics have been used for quality control. We required that more than 90% of each exome had 10× coverage and more than 80% had 20× coverage. Short read sequences were then aligned to hg19 with BWA v0.7.8, duplicate reads were removed with PicardTools MarkDuplicates. Reads with a global quality under 30 or a mapping quality under 20 were excluded from the analysis. Variants were predicted using FreeBayes and GATK^42^ with a minimum of 10 reads covering the position. VEP (using RefSeq and Ensembl 91) was used to annotate the variants. We used the GEMINI^43^ framework that automatically integrates the VCF file into a database for exploring genetic variant for disease and population genetics. Genetic variants were analyzed using GRAVITY, a Cytoscape plugin that we designed for visualizing WES results using Protein-Protein Interaction networks (http://gravity.pasteur.fr/). Since WES does not detect the *FMR1* amplification, 33 individuals with autism were tested for Fragile-X syndrome using the AmplideX^TM^ *FMR1* PCR kit from Theradiag. No individual were carrier of a “pre-mutation” or “full-mutation” of CGG repeats in the 5’ UTR region of the fragile X mental retardation-1 (*FMR1*) gene.

### Copy-number variants (CNVs)

CNVs were identified from both SNP genotyping and WES data. Quality controls were the following: call rate > 0.99, standard deviation of the Log R ratio < 0.35, standard deviation of the B allele frequency < 0.08 and absolute value of the wave factor < 0.05. CNVs were detected by both PennCNV and QuantiSNP algorithms using the following filters: > = 3 consecutive probes, CNV size > 1 kb and CNV detection confidence score > = 15. CNV detections from PennCNV and QuantiSNP were merged using CNVision.^44^ CNVs with CNVision confidence score < 30, CNV size < 50 kb, overlap > 50% with segmental duplication or known large assembly gaps (greater than 150 kb) or copy number = 2 in pseudo autosomal regions (PARS) in males were filtered out. CNV annotations were performed using ANNOVAR^45^ and CNV frequencies in Faroese and in database of genomic variant cohorts (DGV, http://dgv.tcag.ca/dgv/app/home) were assessed using in house python scripts based on reciprocal overlap in size > = 80%. CNV calling from Illumina genotyping data was used only for segregation analysis across families. We also detected CNVs from the WES sequencing data using the XHMM software.^46^ CNVs with QSOME score < 90, number of targets < 5, or overlap > 50% with segmental duplication or known large assembly gaps (greater than 150 kb) were filtered out. CNV annotations were performed using ANNOVAR^45^ and CNV frequencies in Faroese were assessed using in house python scripts based on reciprocal target overlap ≥50% and using only independent cases and controls (*n* = 143). For the burden analysis of rare CNVs, only XHMM CNV calling from WES data was used and CNVs with frequency > 0.01 were filtered out. De novo and inherited CNVs were validated by visual inspection using SnipPeep (http://snippeep.sourceforge.net/).

### Gene-set lists and prioritization of variants

Three gene-set lists were used: (i) “SFARI genes” (*n* = 990) that includes genes implicated in autism^15^ (Simons Foundation Autism Research Initiative gene database–https://gene.sfari.org/); (ii) “pLI > 0.9 genes” that includes genes with strong probability of being loss-of function intolerant (*n* = 3230); (iii) “Brain genes” that includes genes specifically or strongly expressed (above 1 Standard Deviation) in fetal or adult human brain using data from Su et al. (*n* = 3591).^47^

A combination of approaches was used to prioritize the genes and to estimate the deleterious effect of a variant. We prioritized genes using gene sets (SFARI genes, pLI > = 0.9 and Brain genes). We prioritized Likely Gene Disruptive (LGD) variants (stopgains, splice site variants, frameshift indels) over missense variants or synonymous variants. Additionally, we used the CADD score^14^ (a CADD > = 30 means that the variants belong to the 0.1% most deleterious variants) to assess the deleterious effect of missense variants. Minor allele frequency (MAF) was estimated in the general population from the gnomAD database. In order to filter out common variants that was not listed in gnomAD, we also excluded variants that were present in more than 15% of our Faroese control cohort. For the detection of deleterious homozygous variants, we kept only LGD and MIS30 with MAF < 1%.

### Burden analysis

Rare variant association studies (MAF < 5%) were performed using EPACTS v3.2.6 (https://genome.sph.umich.edu/wiki/EPACTS). Prior to association analysis, variants identified by WES were filtered using VCFtools (http://vcftools.sourceforge.net/man_latest.html) with the following metrics: minimum genotyping quality ≥30, min depth of coverage ≥ 10, maximum of missing data ≤ 10, only bi-allelic sites and no site failing Hardy Weinberg equilibrium test (*p* < 10^−6^). The annotation of the variants was done using EPACTS and the variants included in the Gene-wise association analyses were non-synonymous, essential splice site, normal splice site, start loss, stop loss and stop gain variants. Logistic Score Test (“b.score” in S6 Table) was used to test single variant association (*n*_Cases_ = 36; *n*_Controls_ = 107; *n*_Variants_ = 155,284). For Gene-wise tests, we used two approaches (including *n*_Cases_ = 36; *n*_Controls_ = 107 and *n*_groups_ = 15,005): (i) collapsing burden test using EMMAX (Efficient Mixed Model Association eXpedited,^48^ “CMC-EMMAX” in S4 Table) and (ii) Optimal SNP-set sequence Kernel Association Test (“SKAT-O” in S5 Table). The advantage of the CMC-EMMAX is that this test is accounting for population structure and high relatedness between individual (based on kinship matrix). The advantage of SKAT is that this test is particularly powerful in the presence of protective and deleterious variants and null variants. For both Gene-wise tests, a 10^−6 ^≤ MAF ≤ 0.05 was used.

### Statistical power of the analyses

All the statistical tests performed were one-sided since the objectives were to identify some enrichments in autism participants. Achieved power and necessary effect size (using Cohen’s *d*) to achieve a power of 0.8 was computed using G*Power (http://www.gpower.hhu.de/). For CNVs (Fig. 2), given our sample size, the post-hoc achieved power is 0.65 for CNV Loss in all genes, and 0.93 for CNV Gain in all genes. The details on subsets of genes are indicated in S12 Table and the sensitivity to detect effect sizes at a statistical power of 0.8 is indicated in S13 Table. For inbreeding coefficient (Fig. 3a), an effect-size of *d* = 0.43 was observed. Given our sample size, the post-hoc achieved power is 0.74. To achieve a power of 0.8, an effect-size of *d* = 0.47 was needed. For LGD + MIS30 rare homozygous variants (Fig. 3b), the observed effect-size is *d* = 0.68. Given our sample size, the post-hoc achieved power is 0.96. To achieve a power of 0.8, an effect-size of *d* = 0.49 was needed. For GPS (Fig. 4a), the observed effect-size is *d* = 0.45. Given our sample size, the post-hoc achieved power is 0.77. To achieve a power of 0.8, an effect-size of *d* = 0.47 was needed. For GPS between individuals with and without ID (Fig. 4b), the observed effect-size is *d* = 0.80. Given our sample size, the post-hoc achieved power is 0.70. To achieve a power of 0.8, an effect-size of *d* = 0.92 was needed. For enrichment in de novo events for SNVs (Section “Contribution of de novo variants” of the manuscript), given our sample size, an effect size of *d* = 0.60 was required to achieve a power of 0.8.

## Supplementary information

Supplementary Tables

S1 Appendix

## Data Availability

The authors declare that all data supporting the findings of this study are available within the paper and its supplementary information files.

## **Supplementary information**

**Supplementary information** accompanies the paper on the *npj Genomic Medicine* website (10.1038/s41525-018-0075-2).
