# Supplementary material for: Both rare and common genetic variants contribute to autism in the Faroe Islands
Source: NPJ Genom Med. 2019 Jan 21;4:1. doi: 10.1038/s41525-018-0075-2 (PMC6341098; doi:10.1038/s41525-018-0075-2)
Supplement: Supplementary file 2 — S1 Appendix [file 41525_2018_75_MOESM2_ESM.docx]

### S1 appendix

## Material and Clinical notes

# Material

##### Patients

All individuals with autism in this study were recruited from an epidemiological cohort through a two-phase screening and diagnostic process targeting all children born in the 10-year period from 1985 through 1994 and living in the Faroe Islands in 2002 (7-16 years, n=7,689 children) and 2009 (15-24 years, n= 7,128 children) (7)^,^(9). The total number of children diagnosed with autism was 67 which corresponds to an autism prevalence of 0.94%. Among the individuals with autism, 23% were diagnosed with childhood autism, 56% with Asperger syndrome and 21% with atypical autism. There were 49 males (73.1%) and 18 females (26.9%). DNA was available for 36 individuals with autism including 11 diagnosed with childhood autism (31%), 17 with Asperger syndrome (47%), and 8 with atypical autism (22%). There were 28 males (78%) and 8 females (22%). The non-autism controls were recruited by issuing an invitation with information on the study to all pupils at the high school level in winter 2008-2009. The schools invited are in Eysturoy, Suduroy and Torshavn. The age of the invited was from 14-24 years. For those under 18 years a letter was sent to the parents that could sign the consent for their children.

##### Screening and diagnosis

In 2002, screening included the use of the Autism Spectrum Screening Questionnaire (ASSQ). Screen-positive children were thoroughly examined via Diagnostic Interview for Social and Communication Disorder (DISCO-10 in 2002 and DISCO-11 in 2009) of one or both parents and the Wechsler Intelligence Scale for Children – 3^r^ edition (WISC) or Wechsler Adult Intelligence Scale – Revised (WAIS). Whenever overall and verbal abilities allowed it possible, children were also interviewed in an unstructured/semistructured manner about interests and skills patterns, peer relations, family relationships and about formal general information knowledge. The following diagnostic criteria used when making clinical diagnoses were (a) ICD-10 criteria for childhood autism/autistic disorder; (b) Gillberg criteria for Asperger syndrome; (c) ICD-10 criteria for atypical autism with the added requirement that a case thus diagnosed could not meet full criteria for childhood autism or Asperger syndrome; and (d) ICD-10 criteria for disintegrative disorder.

The majority of children in the atypical autism and Asperger syndrome groups had been tested with the WISC-R. Those with childhood autism had usually been tested on other tests. In those intellectually low-functioning individuals for whom no test was available, IQ was estimated on the basis of the Vineland developmental portion that is part of the DISCO-interview.

##### 1000 Genomes populations

1000 Genomes project studied 26 different populations from many different locations around the globe (<http://www.internationalgenome.org/category/population/>). These populations have been divided in five super populations: (1) *African (AFR)* including Yoruba in Ibadan – Nigeria (YRI, n = 108), Luhya in Webuye – Kenya (LWK, n = 99), Gambian in Western Divisions in the Gambia (GWD, n = 113), Mende in Sierra Leone (MSL, n = 85), Esan in Nigeria (ESN, n = 99), Americans of African Ancestry in SW USA (ASW, n = 61), African Caribbeans in Barbados (ACB, n = 96); (2) *Ad Mixed American (AMR)* including Mexican Ancestry from Los Angeles USA (MXL, n = 64), Puerto Ricans from Puerto Rico (PUR, n = 104), Colombians from Medellin – Colombia (CLM, n = 94), Peruvians from Lima – Peru (PEL, n = 85); (3) *East Asian (EAS)* including Han Chinese in Beijing – China (CHB, n = 103), Japanese in Tokyo – Japan (JPT, n = 104), Southern Han Chinese (CHS, n = 105), Chinese Dai in Xishuangbanna – China (CDX, n = 93), Kinh in Ho Chi Minh City – Vietnam (KHV, n = 99); (4) *European (EUR)* including Utah Residents (CEPH) with Northern and Western European Ancestry (CEU, n = 99), Toscani in Italia (TSI, n = 107), Finnish in Finland (FIN, n = 99), British in England and Scotland (GBR, n = 91), Iberian Population in Spain (IBS, n = 107); (5) *South Asian (SAS)* including Gujarati Indian from Houston – Texas (GIH, n = 103), Punjabi from Lahore – Pakistan (PJL, n = 96), Bengali from Bangladesh (BEB, n = 86), Sri Lankan Tamil from the UK (STU, n = 102), Indian Telugu from the UK (ITU, n = 102).

# Clinical Notes

#### PN400100

PN400100 is a male diagnosed with Asperger syndrome (ADOS=14; FSIQ=102).

#### PN400102

PN400102 is a male diagnosed with atypical autism without ID (ADOS=13, FSIQ=97, PIQ=108, VIQ=88). He is carrying a *de novo* variant (p.R485C ) in *PLA2G4A.* This gene codes for the cytosolic phospholipase A2α that catalyzes the hydrolysis of membrane phospholipids to produce arachidonic acid. Mice lacking *Pla2g4a* display abnormalities in neuronal maturation (narrow synaptic cleft) and long-term potentiation. He also carries a maternally inherited p.L888* frameshift variant of the glutamate receptor *GRIK2*, a gene previously found associated with ASD.

#### PN400103

PN400103 is a male diagnosed with Asperger syndrome (ADOS=4, FSIQ=84, PIQ=100, VIQ=73). The patient is carrying a maternally inherited exonic deletion of 58.9 kb including the SFARI gene *IMMP2L*.

#### PN400104

PN400104 is a male diagnosed with atypical autism with ID (ADOS=5, FSIQ=60, PIQ=74, VIQ=61). He has nystagmus. We had no relatives for this individual.

**P**N400108

PN400108 is a male diagnosed with Asperger syndrome (ADOS=15; FSIQ=93). He had a Wilm's tumor (tumor of the kidney) and surgery, radiation therapy and chemotherapy. He is carrying two deleterious variants affecting a SFARI gene, a paternally inherited *BRCA2* stop variant (p.K3326*) and a paternally inherited donor splicing site variant of *ASMT*, the last enzyme of the melatonin synthesis pathway.

#### PN400111

PN400111 is a male diagnosed with Asperger syndrome (ADOS=11; FSIQ=76).

#### PN400113

PN400113 is a male diagnosed with atypical autism (ADOS=2; FSIQ=102). He was a big baby: 60 cm at birth. He is carrying a paternally transmitted stop mutation of the SFARI gene *BRCA2* (p.K3326*).

#### PN400114

PN400114 is a male diagnosed with atypical autism (ADOS=13; FSIQ=73).

PN400115
PN400115 is a male diagnosed with atypical autism (ADOS=22) and severe ID. He has a *de novo* 425.5 kb deletion removing the six first exons of the *NRXN1α*. He has a congenital torticollis and dental carries.

#### PN400116

PN400116 is a male diagnosed with autism and ID (ADOS=15; FSIQ=40). He has cholesteatoma, an abnormal growth of skin in the middle ear behind the eardrum. He is also diagnosed with Tourette syndrome. He is carrying a maternally transmitted splice donor variant of the SFARI gene *YY1* coding for a transcription factor.

#### PN400117

PN400117 is a male diagnosed with Asperger syndrome. He has tics and gynecomastia. He is carrying a *de novo* variant of *KALRN*, a gene involved in synaptic plasticity and homologous to *TRIO*, a gene previously found mutated in ASD. The *KALRN* *de novo* variant (p.N2024D) is predicted deleterious, affects a key amino acid of the GEF domain conserved through evolution.

#### PN400118

PN400118 is a male diagnosed with autism and severe ID (ADOS=22).

#### PN400119

PN400119 is a female diagnosed with childhood autism (ADOS=15, FSIQ=76, PIQ=72, VIQ=80). She has cerebellum atrophy and her mother might have vitamin D. She is carrying a paternally inherited deletion of 248 kb including all exons of *NINL*, a SFARI gene highly express in the cerebellum and coding for protein involved in microtubule organization. The patient is also carrying a private paternally inherited frameshift variant (p.G521Vfs*39) of *ROBO1*, a gene coding for a receptor from the neural cell adhesion molecule that plays a key role in an axon guidance in the brain.

#### PN400121

PN400121 is a female diagnosed with childhood autism (ADOS=22) and severe ID. She was born premature (1.5 kg birth weight) and presented high blood pressure medicine. She is carrying a rare maternally inherited stop variant of the SFARI gene *RBMS3* (p.Q393*) coding for the RNA binding motif single stranded interacting protein 3.

#### PN400124

PN400124 is a male diagnosed with autism and severe ID (ADOS=15). He was born highly premature (27 weeks, 700g at birth) with diplegia spastic degree I (paralysis affecting symmetrical parts of the body). He also has multiple medical conditions including retinopathy, lung infections, necrotizing enterocolitis, cardiac problems and potentially epilepsy.

#### PN400125

PN400125 is a male with a severe ID and childhood autism (ADOS=19) carrying a 91.4 kb deletion removing all exons of *ADNP*. *ADNP* loss of function mutations are usually *de novo*, but, for this patient, we cannot confirm the *de novo* status since we had no DNA from the father. The patient has hypotonia, narrow occiput, small and low ears, epilepsy in the first years of life but then stopped, strabismus, dental caries and otitis.

#### PN400127

PN400127 is a male diagnosed with autism and severe ID (ADOS=21). He developed ataxia after hitting his head at 11 years old. He also has hypotonia.

#### PN400128

PN400128 is a male diagnosed with Asperger syndrome (ADOS=7). He is carrying a paternally inherited frameshift variant in the SFARI gene *ERBB4* (p.M914Ffs*20) coding for the erb-b2 receptor tyrosine kinase 4.

#### PN400129

PN400129 is a male who has a trisomy of chromosome 21 and is diagnosed with childhood autism (ADOS=22) and Down syndrome. He has severe ID, ventricular septum defect, scoliosis and dental caries.

#### PN400132

PN400132 is a male diagnosed with Asperger syndrome (ADOS=8; FSIQ=90). He is carrying a paternally transmitted frameshift variant of the SFARI gene *PPP2R1B* (p.V115Cfs*3). He has a posterior dislocation of the hip.

#### PN400133

PN400133 is a male diagnosed with Asperger syndrome (ADOS=15; FSIQ=86). He suffers from congenital vesico-uretero-renalis reflux and hydronephrosis (a kidney swells disorder due to the failure of normal drainage of urine from the kidney to the bladder).

#### PN400137

PN400137 is a male diagnosed with Asperger syndrome (ADOS=9; FSIQ=114; PIQ=108, VIQ=116). He is carrying two *de novo* variants affecting *RIMS4* (p.Tyr205*) and *GC* (p.Tyr186Ser). *RIMS4* codes for a presynaptic proteins that plays a key role for dendritic and axonal morphogenesis. The *GC* variant alters a conserved region of the Vitamin D-Binding Protein, a key actor for vitamin D transport and homeostasis.

PN400144
PN400144 is a male diagnosed with Asperger syndrome (ADOS=17; FSIQ=88; PIQ=104, VIQ=74). He has epilepsy with abnormal EEG in the temporal region. He is carrying a frameshift variant (p.T1169TX) of *CAD* that could be *de novo* since we had no relatives for this individual. *CAD* codes for an enzyme of the pyrimidine biosynthesis previously associated with early onset epileptic encephalopathy (MIM # 616457). He also carries a splice site variant affecting ASMT, the last protein of the melatonin pathway previously found mutated in patients with ASD.

#### PN400166

PN400166 is a male diagnosed with atypical autism (ADOS=9, FSIQ=78, PIQ=102, VIQ=63). He is a collector and has interest in computers. He is carrying a homozygous *PREX2* variant (p.D312N). *PREX2* codes for a RAC1 guanine nucleotide exchange factor (GEF), is a binding partner of PTEN, a component of the PI3K pathway and has strong homology with PREX1, a SFARI gene. He also carries a paternally inherited p.L888* frameshift variant of the glutamate receptor *GRIK2*, previously found associated with ASD.

#### PN400179

PN400179 is a male diagnosed with Asperger syndrome (ADOS=13; FSIQ=102). He had orthopedic surgery. He is carrying LGD variant in three SFARI genes: a variant in the protein phosphatase 2 scaffold subunit Abeta gene *PPP2R1B* (p.V115Cfs*3), a variant in the complement component 4B gene *C4B* (p.Y1229*), and a variant in the calpain 12 gene *CAPN12* (p.S481CAPTAX). These three variants were not inherited from the father and no DNA from the mother was available.

#### PN400182

PN400182 a male diagnosed with Asperger syndrome (ADOS=9; FSIQ=94).

#### PN400528

PN400528 is a female who is diagnosed with Asperger syndrome (FSIQ=85; PIQ=97; FSIQ=78; ADOS=18). She has primary diurnal enuresis and she is hypersensitive to sound. She showed high inbreeding coefficient (F = 0.041) and carries two deleterious homozygous mutations. An homozygous damaging missense mutation (p.R562L) affects a conserved residue in the cytoplasmic domain of the synaptic adhesion molecule KIRREL3 listed in SFARI and previously associated with neurodevelopmental disorders. She is also homozygous for another deleterious mutation (p.N687K) affecting *TECTA*, a SFARI gene associated with autism and deafness.

#### PN400530

PN400530 is a male diagnosed with atypical autism, ADHD and ID, but no hearing problems (ADOS=4; FSIQ=50; PIQ=67, VIQ=54). He is carrying a homozygous splice acceptor variant affecting *P2RX2* that codes for a cation channel receptor that mediates excitatory postsynaptic responses in sensory neurons. This gene was previously associated with autosomal dominant deafness (MIM #608224).

#### PN400531

PN400531 is a female diagnosed with atypical autism and ID (ADOS=20; FSIQ=37). She suffers from epilepsy (generalized paroxysms of spikes with an irregular frequency of 3–4 Hz).

#### PN400532

PN400532 is a female diagnosed with Asperger syndrome (ADOS=13, FSIQ=78, PIQ=82, VIQ=79). She is carrying a *de novo MECP2* missense (p.R103W) variant predicted as deleterious and within the methyl binding domain of the protein. However, the same variant was observed in three individuals (two females and one male) among the > 90,000 individuals from gnomAD. Therefore, the causative effect of this mutations is not clear. The same patient also carried a *de novo KIF17* variant (p.T306M). *KIF17* codes for a dendrite-specific synaptic molecular motor that transports N-methyl-D-aspartate (NMDA) receptor subunit-2Bcontaining vesicles to neuronal dendrites. The patient displays an absence of corpus callosum, abnormal spikes of both hemispheres on EEG and possibly epilepsy.

#### PN400533

PN400533 is a female diagnosed with atypical autism (ADOS=8, FSIQ=79, PIQ=75, VIQ=86). She carried a *de novo* 2.9 Mb deletion on chromosome 22q11 causing DiGeorge/VeloCardioFacial syndrome. She shows signs of attention deficit and hyperactivity disorder (ADHD). She has flat broad face, high forehead, thick veins visible on forehead, mild asymmetry of the face, right half is prominent, synophrys, low set ears, high nasal bridge, motoric and right half of faceless than left, recurrent infections mainly in the lungs and urinary tract, scoliosis and convulsions at 4-7 days after birth.

#### PN400534

PN400534 is a female diagnosed with atypical autism and ID (ADOS=10; FSIQ=58). She has dental carries. She is carrying a paternally transmitted frameshift variant of the SFARI gene *ICA1* (pV49X) coding for the islet cell autoantigen 1.

#### PN400559

PN400559 is a male diagnosed with Asperger syndrome (FSIQ=92; PIQ=92; VIQ=92). He has sleeping problems, but no vision and hearing problems. He is carrying a 2 Mb paternal inherited duplication on the pseudo-autosomal region 1 including *SHOX* and *ASMT*. He is also compound heterozygous for *USH2A* (p.R4115C inherited from the mother and p.R5031W inherited from the father), a gene involved in hearing and vision loss (MIM # 276901).

#### PN400575

PN400575 is a female diagnosed with Asperger syndrome (ADOS=11; FSIQ=94). She was born premature (1kg at birth).

#### PN400579

PN400579 is a male proband with a high inbreeding coefficient (F = 0.101; parents are first cousins). He is diagnosed with childhood autism without ID, no hearing deficiency and no epilepsy (ADOS=15, FSIQ=101, PIQ=110, VIQ=95). He is homozygous for two variants affecting *CNTNAP2* and *PEX6.* Recessive *CNTNAP2* mutations are associated with Pitt-Hopkins like syndrome 1 (MIM # 610042) and cortical dysplasia-focal epilepsy syndrome (MIM #610042). The *CNTNAP2* p.E680K affects a highly conserved amino acid within the fibrinogen domain of the protein, but is found in the general population although very rare (26 carriers among 138,384 individuals in gnomAD and never at the homozygous state). Recessive *PEX6* mutations are associated with Heimler syndrome 2, a recessive peroxisome disorder characterized by sensorineural hearing loss, amelogenesis imperfecta and nail abnormalities, with or without visual defects (MIM # 616617). The homozygous variant p.R601Q carried by the proband was previously considered pathogenic since it was detected in several independent patients diagnosed with Heimler syndrome 2.

#### PN400587

PN400587 is a male diagnosed with Asperger syndrome (ADOS=12, FSIQ=97, PIQ=97, VIQ=97). He has been in patient care more than 1 year due to psychosis and depression. He is carrying a paternally inherited frameshift variant (p.RK174-175RX) of *RANBP17*, a SFARI gene. *RANBP17* codes for a member of the importin-beta superfamily of nuclear transport receptors and is expressed in the brain (mostly cerebellum).

## Supplementary Figures

**
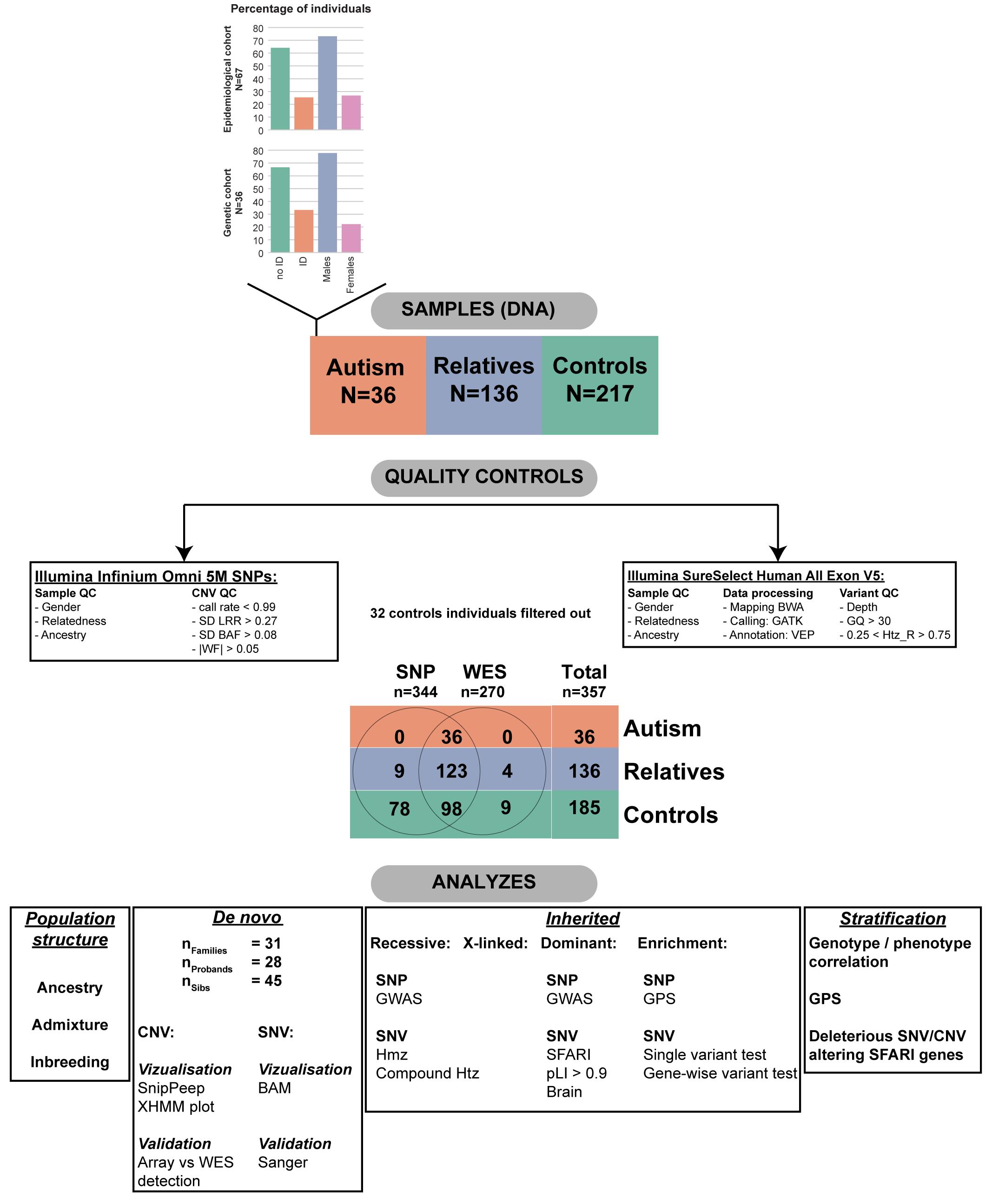
**

S1 Fig. Pipeline of the study. QC, quality control; SD LRR, standard deviation of the Log R ratio; SD BAF, standard deviation of the B allele frequency; |WF|, absolute value of the wave factor; BWA, Burrows-Wheeler Aligner; GATK, Genome Analysis Toolkit; VEP, Variant Effect Predictor; GQ, genotyping quality; Htz_R, heterozygosity ratio; CNV, copy number variant; SNP/V, single nucleotide polymorphism/variation; WES, whole exome sequencing; Hmz, homozygote; Htz, heterozygote; pLI, probability of being loss-of function intolerant; GPS, Genome-wide polygenic score.

**
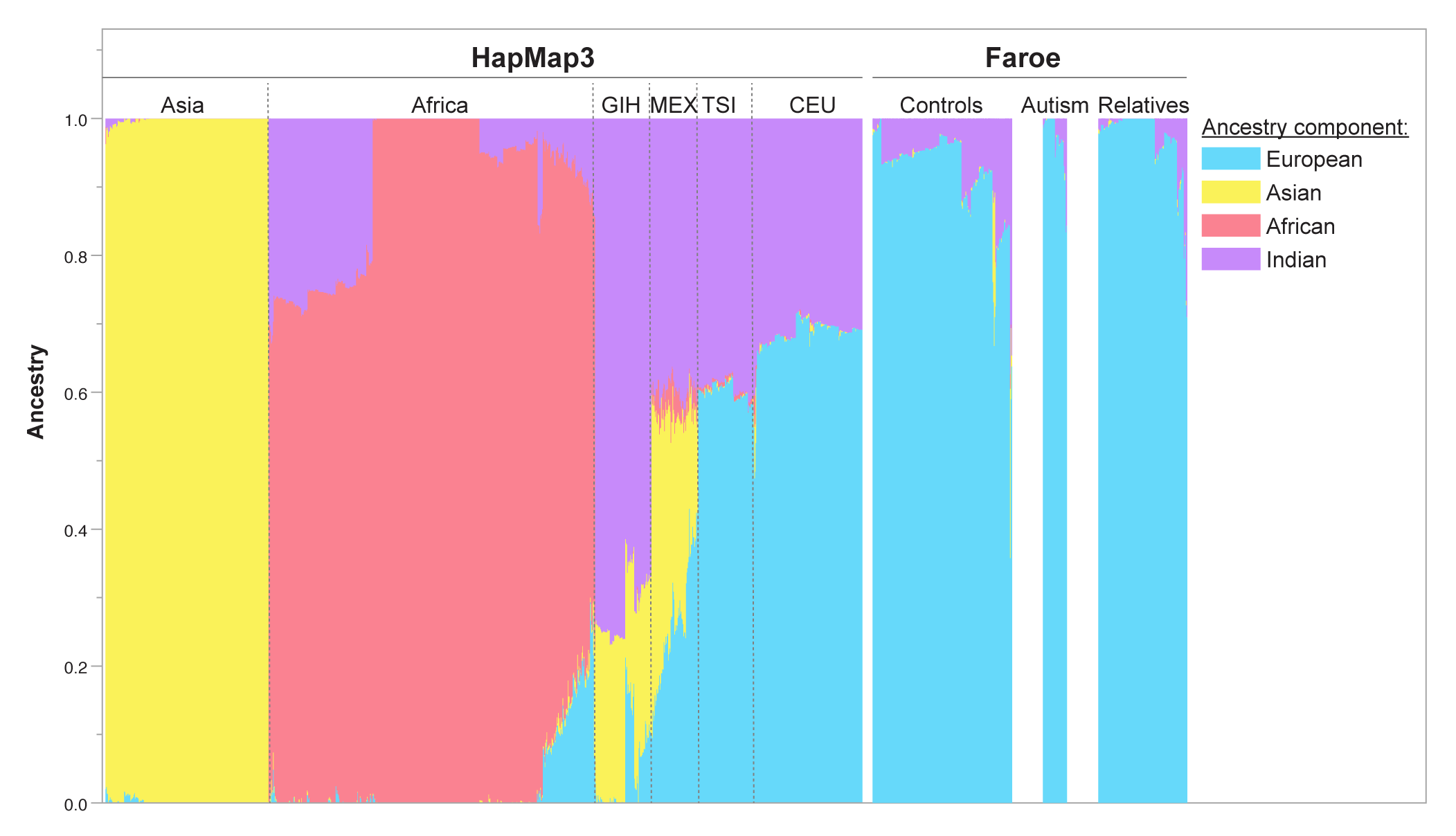
**S2 Fig. Population stratification of the Faroe Islands. Combination of the ethnic genetic background for each individuals of the Faroe and the individuals from HapMap3 using admixture. Legend of HapMap 3 population: Africa (ASW, African ancestry in Southwest USA (n=83); LWK, Luhya in Webuye, Kenya (n=90); MKK, Maasai in Kinyawa, Kenya (n=171); YRI, Yoruba in Ibadan, Nigeria (n=167)); Asia (CHB, Han Chinese in Beijing, China (n=84); CHD, Chinese in Metropolitan Denver, Colorado (n=85); JPT, Japanese in Tokyo, Japan (n=86)); TSI, Toscani in Italia (n=88); CEU, Utah residents with Northern and Western European ancestry from the CEPH collection (n=165); GIH, Gujarati Indians in Houston, Texas (n=88); MXL, Mexican ancestry in Los Angeles (n=77), California.

****S3 Fig. Distribution of the homozygous segment (ROH) length. A. Distribution of the ROH length (KB) in the entire Faroese cohort. B. Histogram of ROH length (KB) within two controls (PN400438 & PN400498) with average overall homozygous segment length, one mother (PN400545) with an accumulation of ROH and one patient (PN400579) with very long ROH. ROH, Runs Of Homozygosity; KB, kilobase; FROH, Inbreeding coefficient.

**
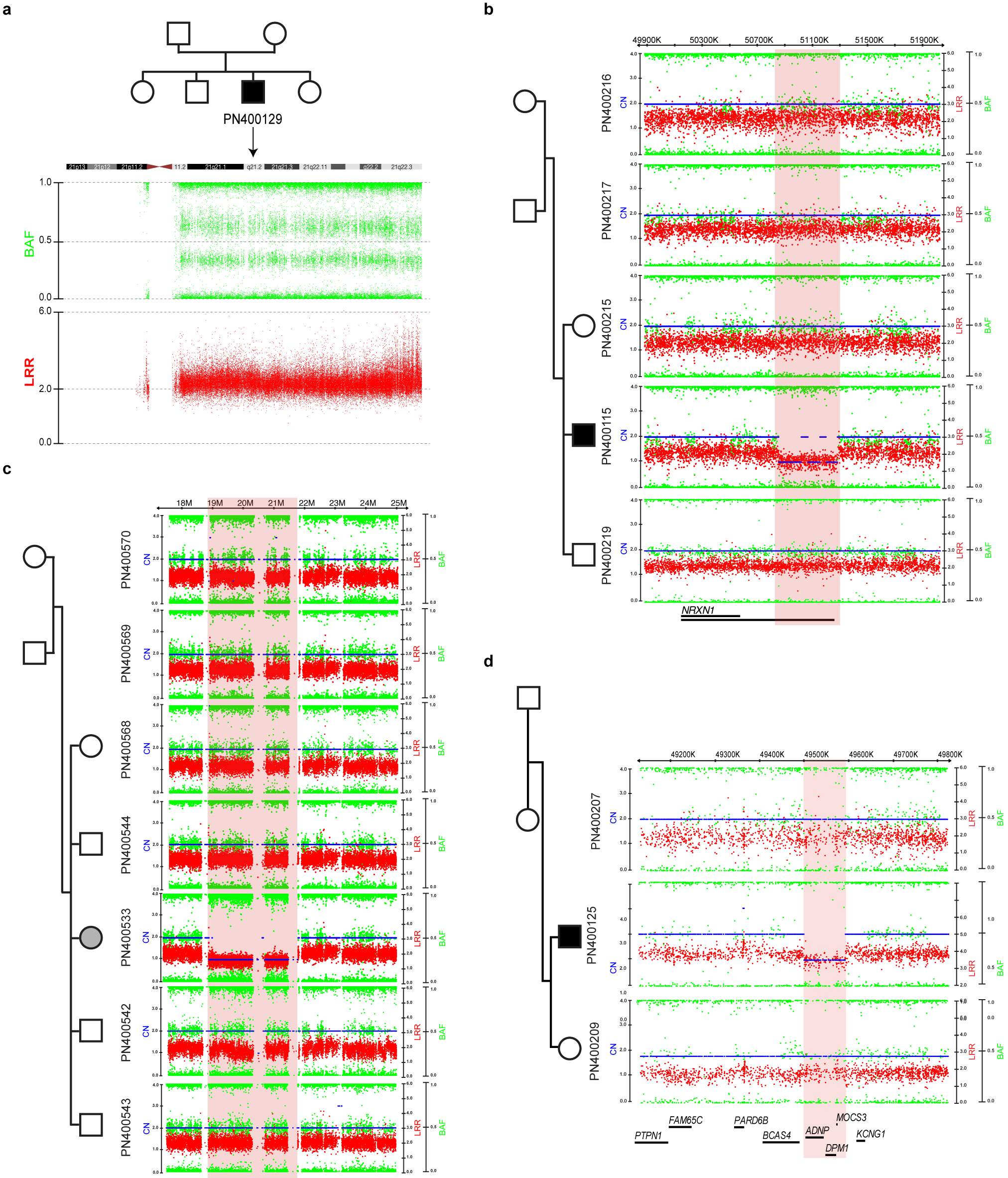
**S4 Fig. CNVs altering genes involved in neurodevelopmental disorders. A. *De novo* Trisomy 21 in patient with autism, ID and Down syndrome. B. *De novo* deletion of 425.5 kb removing the six first exons of the *NRXN1α* in individual with autism. C. *De novo* 2.9 Mb deletion on chromosome 22q11 in individual with autism and 22q11.2 deletion syndrome. D. A 91.4 kb deletion removing all exons of *ADNP* in a male with autism and ID. Each dot shows Log R Ratio (LRR; in red) and B allele frequency (BAF; in green). The copy number (CN) is indicated with a blue line. Patients with ID and patients without ID are represented in black and grey, respectively. ID, Intellectual disability.

****S5 Fig. The *de novo* SNVs in individuals with autism. Sanger sequencing was performed to validate *de novo* SNVs altering *MECP2*, *KIF17* (A), *PLA2G4A* (B), *RIMS4* (C) and *KALRN* (D). Sanger chromatograms are shown for each trios. Individuals with ID and without ID are represented in black and grey, respectively. The position of the SNVs is indicated on the protein and the amino acid alignment of the region throughout several species (Human, Rhesus, Mouse, Dog, Zebrafish) shows the high conservation of the altered amino acids; ID, Intellectual disability.

**
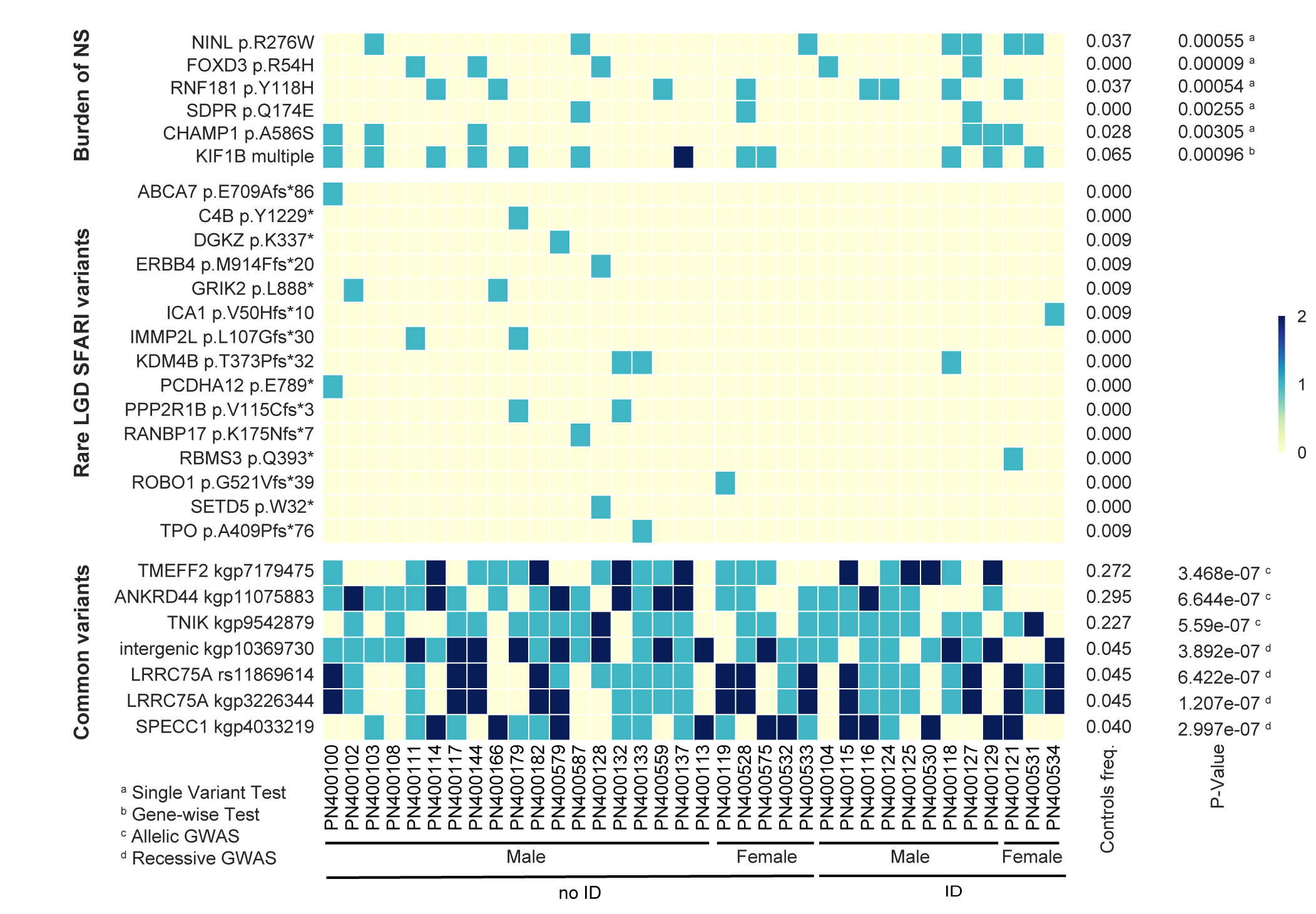
**S6 Fig. Heatmap combining signals obtained from rare and common variant association tests and rare deleterious variants altering SFARI genes throughout Faroese individuals with autism. “Burden of non-synonymous” includes results from SKAT-O and CMC-EMMAX obtained from WES data (see Methods section and S7 fig; p < 10^^-3^). “Common variants” are the top hits of the genome wide association study (GWAS) for both allelic and recessive model obtained from genome-wide genotyping data (p < 10^^-6^). “Rare LGD SFARI variants” are rare likely gene disrupting (LGD) variants altering SFARI genes identified by (MAF < 1% in gnomAD). “The controls freq.” column indicates the proportion of non-ASD Faroese controls carrying the corresponding variant. P-Values are nominal. ID, intellectual disability. NS, non-synonymous variant.

****S7 Fig. Gene-wise association study using the whole exome sequencing data. To test for Gene-wise association, a collapsing burden test using EMMAX “CMC-EMMAX” (A) and optimal SNP-set sequence Kernel Association Test “SKAT-O” (B) were used. The dashed line indicates p-value < 10^^-3^. EMMAX, Efficient Mixed Model Association eXpedited.

****S8 Fig. Results from the Genome Wide Association Study (GWAS) using different models (allelic, Recessive or Dominant). QQ plots and Manhattan plots for allelic, recessive and dominant GWAS are represented in panel A and B, respectively. QQ, quantile-quantile.

**
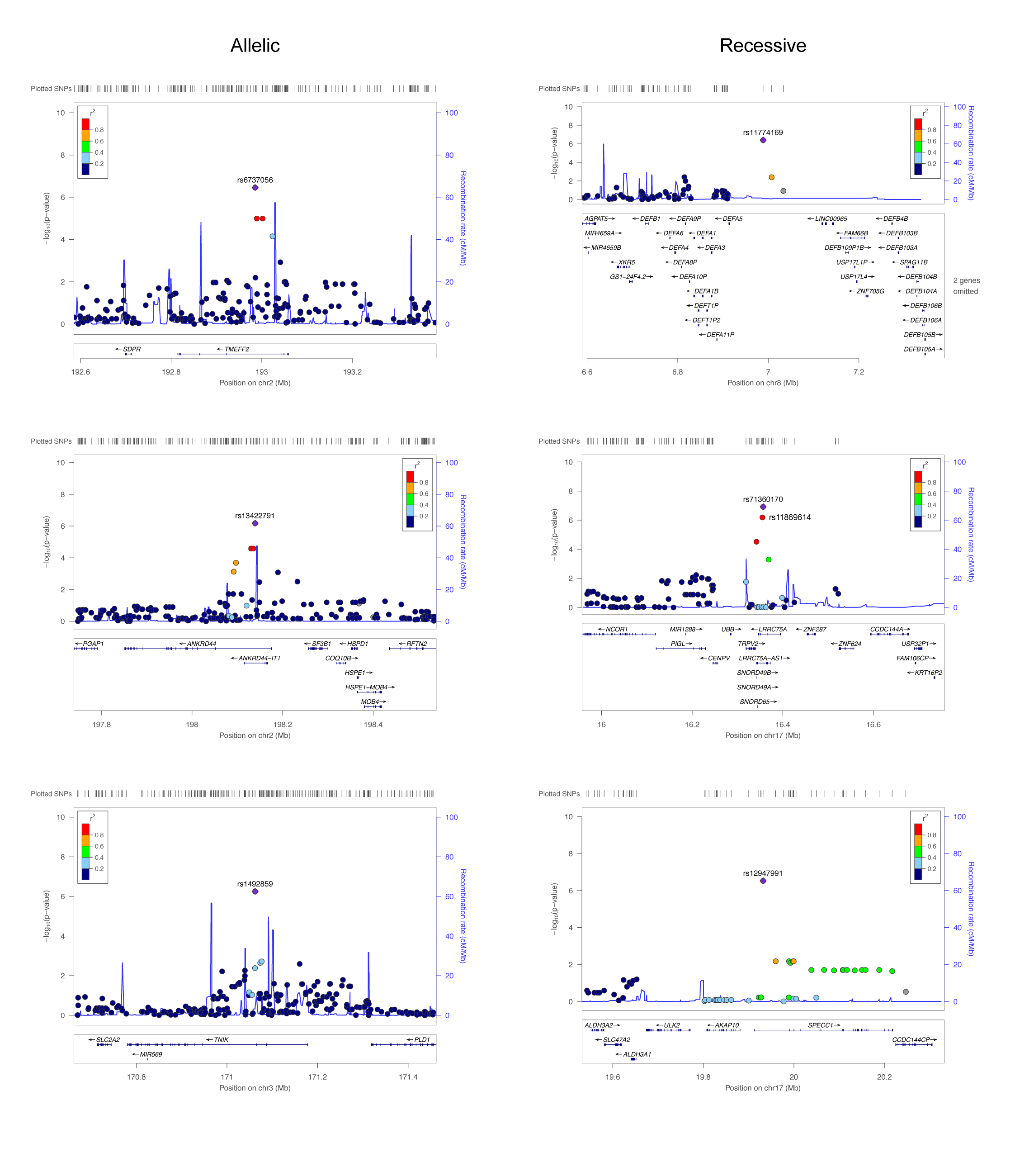
**S9 Fig. Locus zoom of the top hits detected by the GWAS. LocusZoom for regional visualization of the top hits isolated from the allelic and recessive GWAS was used.

**
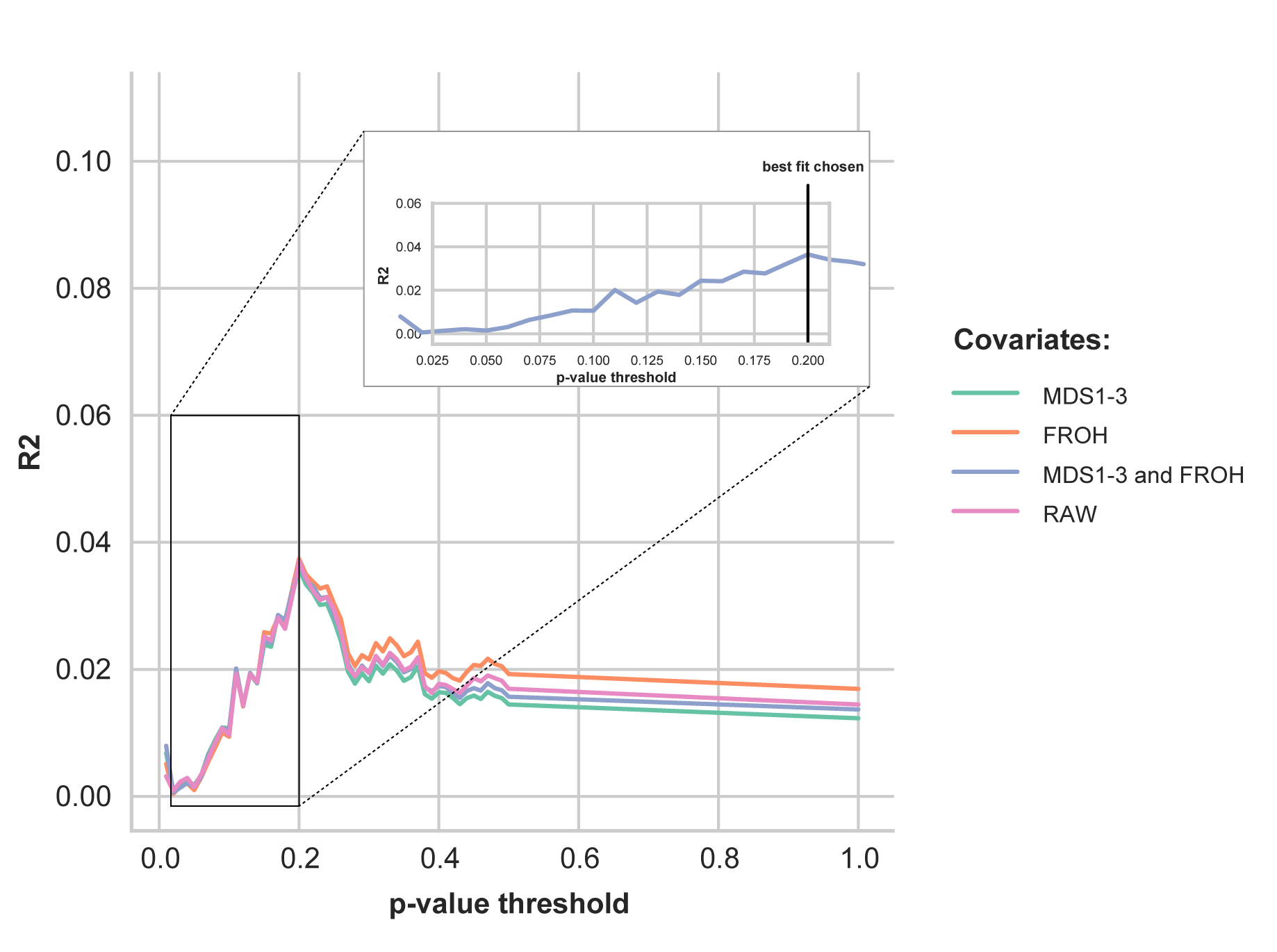
**S10 Fig. Target sample variance explained in function of P-value threshold. The target sample is composed of 212 independent individuals including 36 patients with autism and 176 controls. R2**,** squared correlation coefficient**;** RAW, Faroese data without correction; MDS1-3, Faroese data ancestry-adjusted using C1-3 of the MDS; FROH, Faroese data Inbreeding-adjusted for; MDS1-3 and FROH, Faroese data Ancestry and Inbreeding-adjusted.
